# Adolescent With VACTERL Association Presents With Recurrent Pneumonia

**DOI:** 10.7759/cureus.10365

**Published:** 2020-09-10

**Authors:** Michael Stack, Tamarah Westmoreland

**Affiliations:** 1 Medicine, University of Central Florida College of Medicine, Orlando, USA; 2 Pediatric Surgery, Nemours Children's Hospital, Orlando, USA

**Keywords:** vacterl, tracheoesophageal fistula, tef, h type tef

## Abstract

VACTERL is a condition that includes various anatomic anomalies including vertebral, cardiac, tracheoesophageal fistula (TEF), renal, and limb defects. TEF can be found in up to 80% of patients with the condition. Although TEFs are usually identified early in life, the H-type TEF can be more difficult to detect. We report the case of a 15-year-old male with a previous diagnosis of VACTERL who presented with a history of recurrent pneumonia, chest pain, and asthma and was found to have a previously undetected H-type TEF that was surgically repaired. When evaluating a patient with features of VACTERL, it is important to choose studies that can explore the presence of all associated features. Clinical history and type of imaging utilized can be essential in making a timely diagnosis, especially for H-type TEF.

## Introduction

VACTERL association describes the co-occurrence of several congenital anomalies including vertebral (V), anorectal (A), cardiac (C), tracheoesophageal fistula (TE or TEF), renal (R), and limb (L) abnormalities. These anomalies are usually discovered early in life and are surgically corrected [1]. TEF can be seen in 50-80% of patients with VACTERL association [2]. TEF accounts for approximately one in 3,500 live births and is most often associated with congenital cardiac anomalies. Within this group, esophageal atresia (EA) with TEFs co-occur in 90% of cases and are usually diagnosed and surgically managed in early infancy [3,4]. Patients with TEFs typically present with coughing, drooling, cyanosis, feeding difficulties, and respiratory distress. However, 4% of TEFs occur in isolation without EA, and these are known as the H-type or Type E TEF. This type of TEF can also present with recurrent pneumonia, brief resolved unexplained events, and respiratory symptoms. However, due to the lack of EA, the presentation can be later in infancy with milder symptoms and decreased recognition of the condition clinically. Rarely, H-type TEF can be detected in teenagers and young adults [1,5]. While fluoroscopic esophagram and bronchoscopy are the primary methods to confirm diagnosis, computed tomography (CT) and endoscopy are also utilized as well given certain characteristics of the H-type TEF [6-8]. Alternatively, this form of TEF is often described as the ‘N-type’ because the tract commonly arises from the proximal trachea and inserts distally in the esophagus. This oblique tract can cause diagnostic difficulties and often results in false-negative findings on esophagram and CT, which can be decreased with proper positioning and imaging techniques. The fistula can also remain undetected upon direct visualization via bronchoscopy due to its small size and the presence of mucosal redundancy. The presence of a mucosal flap has also been thought to obscure the fistula intermittently. Initial negative findings with sufficient clinical suspicion may warrant repeated testing with different imaging modalities to confirm a diagnosis [5,9,10].

## Case presentation

A 15-year-old male with a prior diagnosis of VACTERL association presented with respiratory symptoms for several years including recurrent pneumonia, episodes of chest pain, and moderate persistent asthma. At a young age, several anomalies associated with VACTERL were identified and surgically repaired. The patient’s cardiac anomalies included transposition of great vessels, single coronary artery, ventricular septal defect (VSD), and patent ductus arteriosus (PDA). The patient's other anomalies included vertebral body deformity with scoliosis, bilateral cleft hands, and right radial dysplasia. The patient experienced failure to thrive as an infant requiring gastrostomy tube feeding. His medical history also included gastroesophageal reflux disease (GERD) and Nissen fundoplication. The patient had a mild cognitive delay and denied any dysphagia with solids or liquids. The patient reported the ability to belch up large amounts of fluid and food.

**Figure 1:**
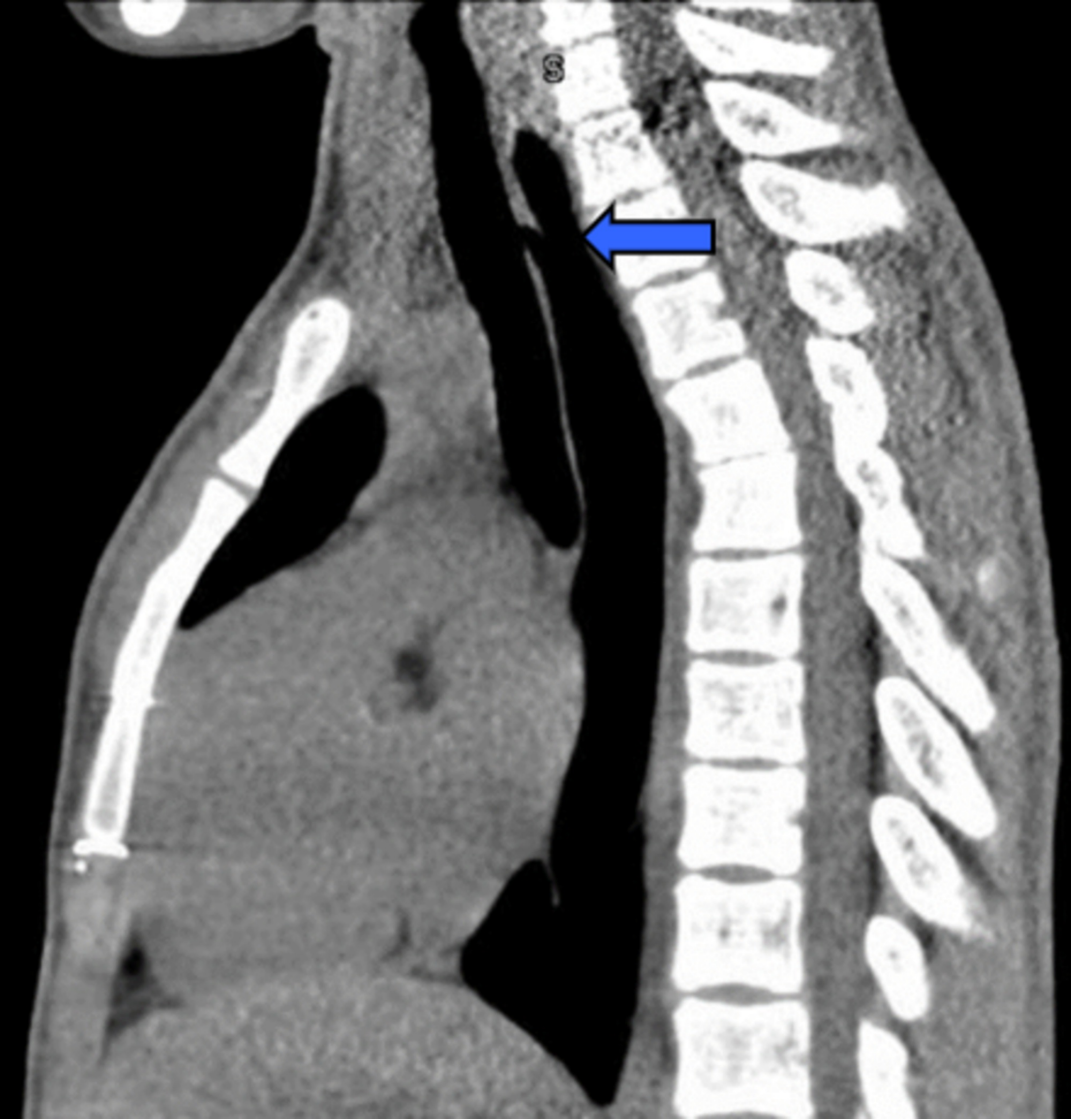
CT scan of chest without contrast, sagittal view, demonstrating TEF (blue arrow). TEF: tracheoesophageal fistula.

A CT scan from another institution showed communication between the trachea and esophagus approximately 2 cm superior to the manubrium along with a dilated esophagus (Figure 1). The TEF was not seen on a previous CT scan. Prior to surgical intervention, a cardiology consult was completed along with an echocardiogram to confirm vessel anatomy. Bronchoscopy and esophagoscopy were performed preoperatively to confirm H-type fistula location and to exclude the presence of a proximal fistula tract. The fistula was noted to be 6 cm distal to the vocal cords and then was cannulated with a Fogarty catheter to aid in localizing the tract intraoperatively. The tissue surrounding the fistula was fibrotic and the tracheal side of the tract measured approximately 1.5 cm in diameter and was larger than the esophageal segment of the tract. Surgical repair was performed via a left cervical approach, and a sternohyoid muscle flap was placed over the repair between the trachea and the esophagus.

**Figure 2:**
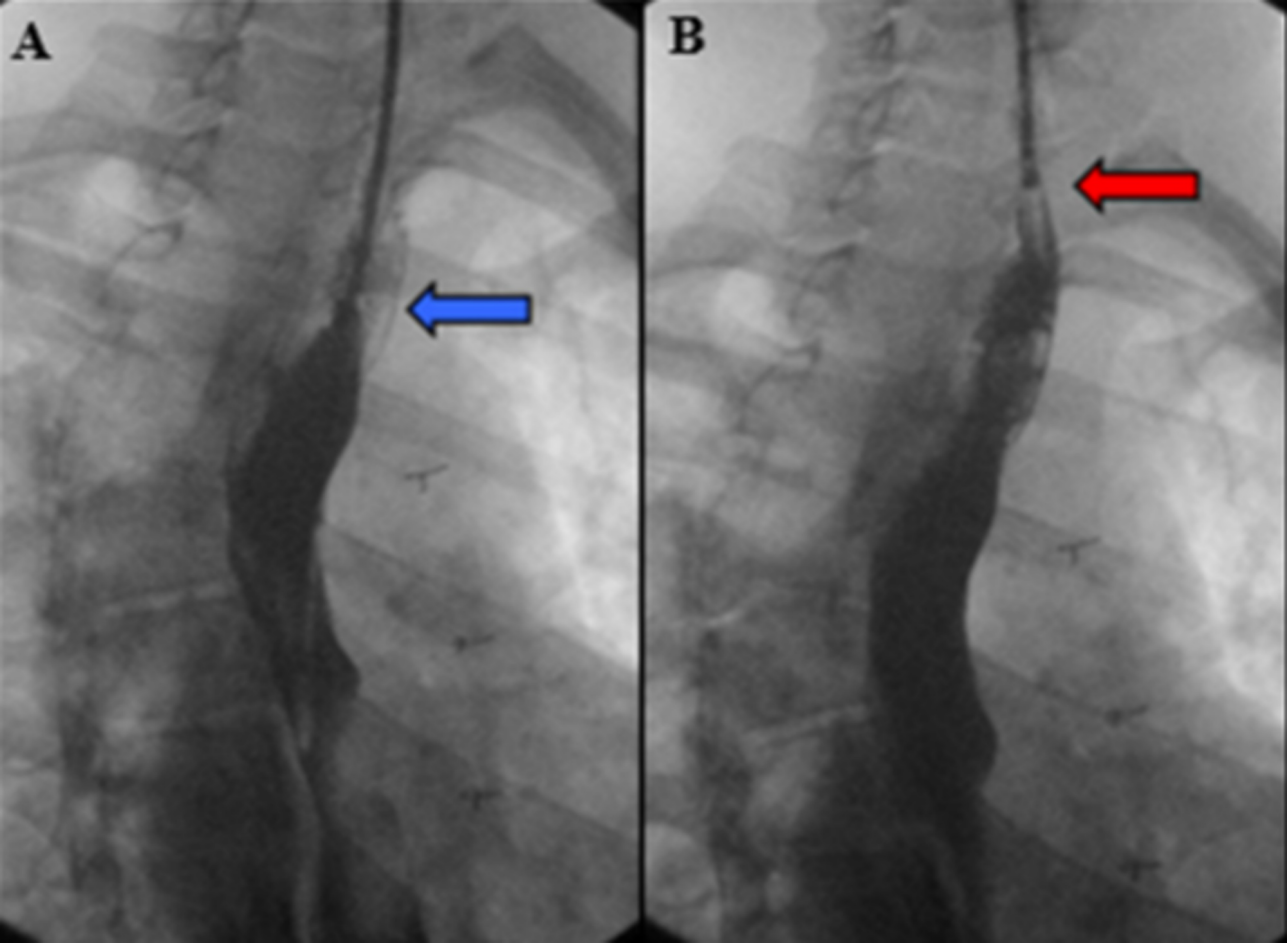
Esophagram, (A) nasogastric tube retracted from just distal to anastomosis (blue arrow), and then (B) proximal to anastomosis (red arrow) with contrast injected at anastomotic site.

The patient was transported to the intensive care unit (ICU) in stable condition and remained intubated overnight with care to avoid excessive suctioning of the endotracheal tube and positive pressure to prevent failure of the repair. The patient was closely followed postoperatively with monitoring for possible expanding hematoma, fever, and respiratory distress. Laryngoscopy was performed upon extubation, which confirmed normal laryngeal vocal cord motion. A contrast swallow study and fluoroscopic esophagogram were performed two days postoperatively demonstrating no leak from the repair site or residual patency of the repaired fistula (Figures 2A and 2B). The patient tolerated a soft mechanical diet and denied hoarseness, change in voice, and difficulty breathing. The postoperative course was without complication and he was discharged on hospital day four. The patient will continue to be monitored in an outpatient clinic.

## Discussion

**Figure 3:**
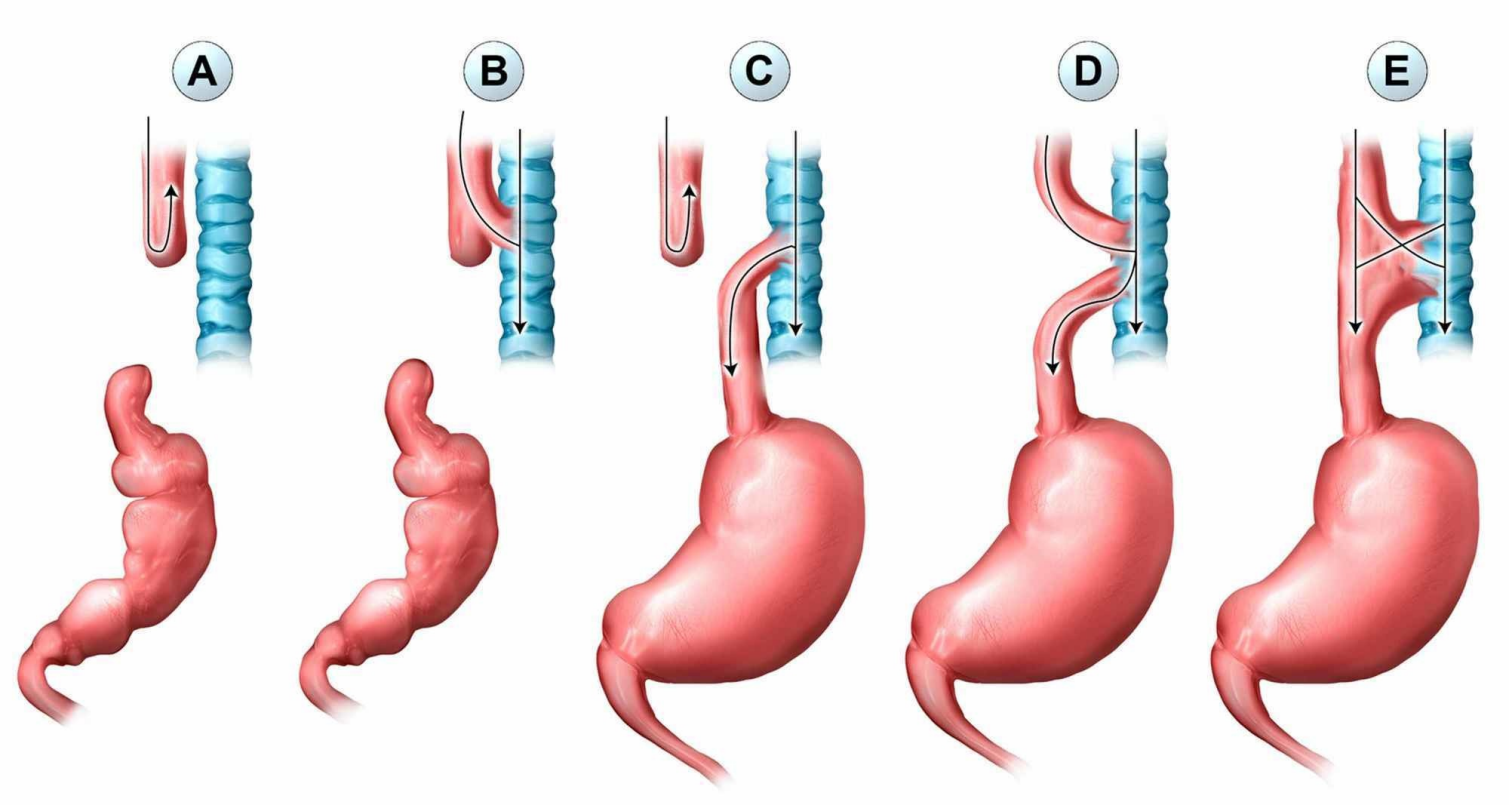
Five types of TEF. Type (A) isolated EA, type (B) EA with proximal TEF, type (C) EA with distal TEF, type (D) EA with proximal and distal TEF, and type (E) isolated TEF (H-type). TEF: tracheoesophageal fistula, EA: esophageal atresia.

Initial symptoms of the H-type TEF can be subtle, posing a challenge for clinicians to detect. The five types of TEF are depicted in Figures 3(A)-3(E). Type E or H-type TEF is distinguished from the other anatomic variants by the absence of EA and typically later clinical presentation. Perhaps the milder symptoms do not raise clinical suspicion enough for further investigation. However, careful attention to the patient’s history can be informative. For example, Wetzler et al. recently described a case report of an adolescent that described an inability to drink from a water fountain without coughing, due to his H-type fistula [11]. Our patient described the ability to reflux quantities of food and liquid on command. Due to our patient’s Nissen procedure, air entering the lower pressure esophagus from the trachea leads to considerable dilation. These unique findings in addition to prior VACTERL diagnosis may increase clinical suspicion for TEF. Delay in diagnosis can lead to increased respiratory complications such as recurrent pneumonia and abscesses. A diagnosis or clinical suspicion of VACTERL should warrant further studies to identify associated anomalies, including echocardiogram, renal ultrasound, and bronchoscopy [1,3]. Over 50% of H-type fistulas can be missed on routine contrast swallow studies [12]. For this reason, a prone Cine tube esophagogram is recommended for better detection of this defect. This is done by slowly withdrawing the catheter while injecting the contrast in the prone position. In the case of our patient, his fistula was large enough to be detected on CT imaging, but smaller defects may not be detected [13]. Interestingly, a forthcoming study utilized end-tidal carbon dioxide measurements during esophagoscopy with insufflation in combination with traditional bronchoscopy and fluoroscopic esophagoscopy to improve diagnostic sensitivity for TEF detection [14].

H-type TEF is technically challenging to repair surgically and many factors must be taken into account for operative planning. Although this practice varies by surgeon or site, a preoperative bronchoscopy can be done to evaluate the upper esophagus to exclude a potential proximal fistula [13]. It is important to accurately localize the fistula and assess for anatomic anomalies, such as a right-sided aortic arch or laryngeal cleft, which may determine the surgical approach [15]. Most patients with H-type fistula are managed with a cervical approach. Careful attention must be paid to surgical anatomy, with emphasis on the recurrent laryngeal nerve. One multicenter review found that 22% of patients who undergo H-type fistula repair experience vocal cord dysfunction postoperatively; 8% leak rate and 3% fistula recurrence. Vocal cord dysfunction due to recurrent laryngeal nerve damage is the most common postoperative complication, leading some to advocate for early routine vocal cord evaluation with laryngoscopy to detect nerve damage [16].

## Conclusions

We present this rare case of an adolescent with VACTERL association and a history of recurrent pneumonia and longstanding feeding and respiratory symptoms. This case highlights the unique symptomatology and multidisciplinary approach to the diagnosis and management of H-type TEF. Owing to the difficulties of detection with diagnostic imaging, a high index of clinical suspicion is often necessary to confirm the diagnosis. Early diagnosis of H-type TEF can be elusive due to its subtle and varied presentation in conjunction with a lack of distinct guidelines for diagnostic workup. Improved knowledge of this uncommon condition will result in a more timely diagnosis for these patients. Prompt and definitive intervention for an H-type TEF will limit further scarring and prevent long-term pulmonary complications.

## References

[1] Solomon BD, Baker LA, Bear KA (2014). An approach to the identification of anomalies and etiologies in neonates with identified or suspected VACTERL (vertebral defects, anal atresia, tracheo-esophageal fistula with esophageal atresia, cardiac anomalies, renal anomalies, and limb anomalies) association. J Pediatr.

[2] Kallen K, Mastroiacovo P, Castilla EE, Robert E, Kallen B (2001). VATER non-random association of congenital malformations: study based on data from four malformation registers. Am J Med Genet.

[3] Spitz L (2006). Esophageal atresia: lessons I have learned in a 40-year experience. J Pediatr Surg.

[4] Shaw-Smith C (2006). Oesophageal atresia, tracheo-oesophageal fistula, and the VACTERL association: review of genetics and epidemiology. J Med Genet.

[5] Brookes JT, Smith MC, Smith RJ, Bauman NM, Manaligod JM, Sandler AD (2007). H-type congenital tracheoesophageal fistula: University of Iowa experience 1985 to 2005. Ann Otol Rhinol Laryngol.

[6] Edelman B, Selvaraj BJ, Joshi M, Patil U, Yarmush J (2019). Anesthesia practice: review of perioperative management of H-type tracheoesophageal fistula. Anesthesiol Res Pract.

[7] Mattei P (2012). Double H-type tracheoesophageal fistulas identified and repaired in 1 operation. J Pediatr Surg.

[8] Klouda TM, Lindholm E, Poletto E, Rani S, Varlotta L, Velasco J (2019). Presentation of an H-type tracheoesophageal fistula in an adolescent male with cystic fibrosis: a case report and review of literature. Clin Imaging.

[9] Risher WH, Arensman RM, Ochsner JL (1990). Congenital bronchoesophageal fistula. Ann Thorac Surg.

[10] Dai J, Pan Z, Wang Q (2018). Experience of diagnosis and treatment of 31 H-type tracheoesophageal fistula in a single clinical center. Pediatr Surg Int.

[11] Wetzler G, Jo I, Breglio K, Kazachkov M (2017). Adolescent presentation of congenital tracheoesophageal fistula. J Pediatr Gastroenterol Nutr.

[12] Harmon C, Coran A (2012). Congenital anomalies of the esophagus. Pediatric Surgery.

[13] Ng J, Antao B, Bartram J, Raghavan A, Shawis R (2006). Diagnostic difficulties in the management of H-type tracheoesophageal fistula. Acta Radiol.

[14] Yasuda JL, Staffa SJ, Ngo PD, Clark SJ, Jennings RW, Manfredi MA (2020). Comparison of detection methods for tracheoesophageal fistulae with a novel method: capnography with CO2 insufflation. J Pediatr Gastroenterol Nutr.

[15] Ko BA, Frederic R, DiTirro PA, Glatleider PA, Applebaum H (2000). Simplified access for division of the low cervical/high thoracic H-type tracheoesophageal fistula. J Pediatr Surg.

[16] Fallon SC, Langer JC, St Peter SD (2017). Congenital H-type tracheoesophageal fistula: a multicenter review of outcomes in a rare disease. J Pediatr Surg.

